# Correlation between hand grip strength and regional muscle mass in older Asian adults: an observational study

**DOI:** 10.1186/s12877-022-02898-8

**Published:** 2022-03-15

**Authors:** Jessica Chan, Yi-Chien Lu, Melissa Min-Szu Yao, Russell Oliver Kosik

**Affiliations:** 1 Postbaccalaureate Prehealth Studies Program, New York University, New York, USA; 2 Department of Radiology, Wan Fang Hospital, Taipei Medical University, 111 Xing Long Road, Section 3, Taipei, 116 Taiwan; 3 Department of Radiology, School of Medicine, College of Medicine, Taipei Medical University, Taipei, Taiwan

**Keywords:** Asian, Hand grip strength, Muscle mass, Older adults, Sarcopenia

## Abstract

**Background:**

Previous research has demonstrated a correlation between hand grip strength (HGS) and muscle strength. This study aims to determine the relationship between HGS and muscle mass in older Asian adults.

**Methods:**

We retrospectively reviewed the dual-energy X-ray absorptiometry (DXA) records of 907 older adults (239 (26.4%) men and 668 (73.6%) women) at one medical institution in Taipei, Taiwan, from January 2019, to December 2020. Average age was 74.80 ± 9.43 and 72.93 ± 9.09 for the males and females respectively. The inclusion criteria were: 1) aged 60 and older, 2) underwent a full-body DXA scan, and 3) performed hand grip measurements. Patients with duplicate results, incomplete records, stroke history, and other neurological diseases were excluded. Regional skeletal muscle mass was measured using DXA. HGS was measured using a Jamar handheld dynamometer.

**Results:**

Total lean muscle mass (kg) averaged 43.63 ± 5.81 and 33.16 ± 4.32 for the males and females respectively. Average HGS (kg) was 28.81 ± 9.87 and 19.19 ± 6.17 for the males and females respectively. In both sexes, HGS and regional muscle mass consistently declined after 60 years of age. The rates of decline per decade in upper and lower extremity muscle mass and HGS were 7.06, 4.95, and 12.30%, respectively, for the males, and 3.36, 4.44, and 12.48%, respectively, for the females. In men, HGS significantly correlated with upper (*r* = 0.576, *p* < 0.001) and lower extremity muscle mass (*r* = 0.532, *p* < 0.001). In women, the correlations between HGS and upper extremity muscle mass (*r* = 0.262, *p* < 0.001) and lower extremity muscle mass (*r* = 0.364, *p <* 0.001) were less strong, though also statistically significant.

**Conclusion:**

Muscle mass and HGS decline with advancing age in both sexes, though the correlation is stronger in men. HGS measurements are an accurate proxy for muscle mass in older Asian adults, particularly in males.

**Supplementary Information:**

The online version contains supplementary material available at 10.1186/s12877-022-02898-8.

## Introduction

Muscle mass and muscle strength are two distinct descriptors of musculature. Mass is a quantitative measure, referring to the muscle fiber hypertrophy or atrophy that occur with muscle growth or loss. Meanwhile, strength describes the force generated by muscle fiber contraction. While the two undoubtedly influence each other, their relationship is complex, and both may be affected by extra-muscular phenomena, such as neurological signaling and certain mechanical factors. Further, as muscle ages, the complexity of the relationship between strength and mass increases, though both have important implications on overall health.

Muscle strength is a strong indicator of mortality and several chronic diseases in older adults. Epidemiological studies have revealed an inverse relationship between muscle strength and all-cause mortality [1–9]. Rantanen et al examined muscle strength as a predictor of mortality in 6040 men over 30 years old. Mortality rates were the highest in men who had the lowest grip strength, and mortality decreased with increasing levels of grip strength [4]. A subsequent study by Fitzgerald et al that included 9105 men and women demonstrated similar findings [10]. Newman et al. further confirmed that reduced muscle strength is a strong and independent predictor of mortality in older adults [6].

Muscle strength and muscle mass both decline with age, though the decline in muscle strength tends to be greater than the decline in muscle mass [11]. Studies conducted across various international populations support this assertion. Results from a recent Polish study showed that the annual losses in muscle strength and function were larger than the loss in appendicular muscle mass in older adults [12]. An Australian study showed that mean grip strength declined with advancing age in older women and that mean grip strength increased with appendicular lean mass [13]. A U.S. study demonstrated that while muscle strength and muscle mass declined with age, loss of strength was greater than loss of muscle mass, and loss of strength varied across sex and race [11]. Results from a multiracial Singaporean study showed that muscle strength decreased overall with age, though there were variations in magnitude across different ethnic groups and between sexes [14].

Hand grip strength (HGS) has been used as a standard for measuring overall muscle strength [15]. Prior research has confirmed an association between grip strength and the strengths of other muscles in both healthy adults and adults afflicted with disease [15]. In another study that included 384 children, adolescents, and young adults, Wind et al. found that there was a strong correlation between hand grip strength and total muscle strength [16].

A few reports have suggested a potential link between HGS and regional muscle mass in older Asian adults [14, 17, 18], though none of these studies directly investigated this relationship. The primary aim of the Singaporean study was to determine the range of normal HGS scores in older adults and not to investigate the relationship between HGS scores and muscle mass, however, regression analysis did reveal an association between HGS and upper arm circumference in males as well as weight and waist circumference in females [14]. While such associations potentially imply an association between HGS and muscle mass, they could also be explained by increased upper extremity fat and abdominal fat in males and females, respectively. A 2020 Korean study attempted to determine the relationship between appendicular muscle mass and frailty [17]. The researchers identified HGS as a mediating variable between appendicular muscle mass and frailty, suggesting a link between HGS and appendicular muscle mass. However, as this was not the primary aim of the study, the study’s ability to detect such a link was limited. Further, the study failed to examine axial muscle mass and therefore the relationship between HGS and total muscle mass. Finally, a prospective Japanese study followed older Japanese for 12 years, attempting to determine the changes in skeletal muscle mass that would occur [18]. As part of the study, HGS was measured and was found to decrease with age. Muscle mass was also found to decrease with age, suggesting a potential relationship with HGS, though again, the primary aim of the research was not to determine such a relationship. Additionally, muscle mass did not decrease with age uniformly and not at all in certain subgroups, further limiting the study’s ability to establish the relationship between HGS and muscle mass.

There is a dearth of research exploring the relationship between HGS and muscle mass. Specifically, no prior study has directly examined the relationship between HGS and muscle mass in older Asian adults. This study aims to fill the void in the literature by determining whether or not such a relationship exists and how the relationship changes across age and gender.

## Methods

### Study population

This work was approved by the Taipei Medical University-Joint Institutional Review Board (JIRB Number N202104037), and the need for informed consent was waived because of the study’s retrospective nature. All methods were performed in accordance with the relevant guidelines and regulations.

**Fig. 1:**
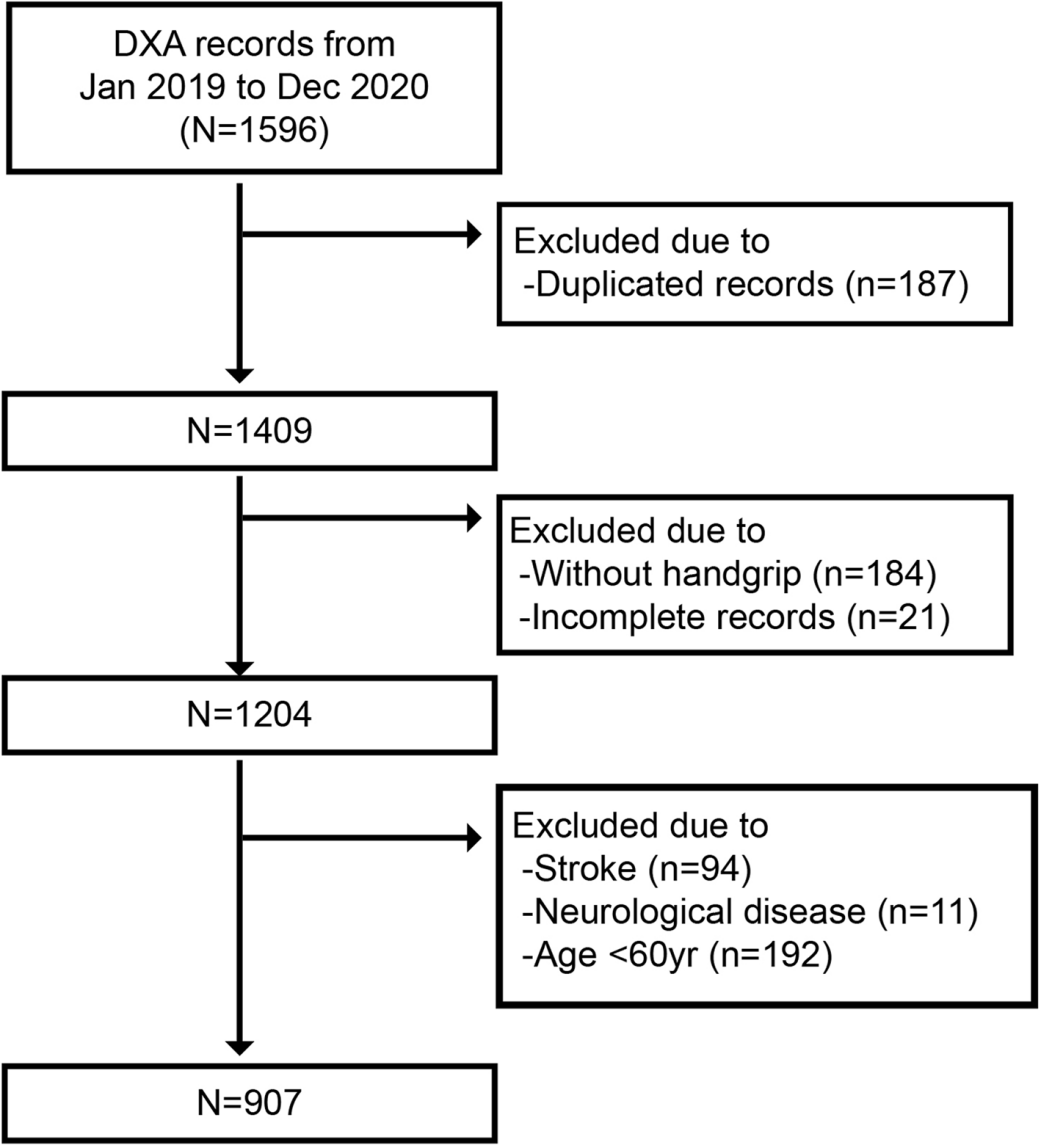
Flow chart indicating the selection of our sample population

We retrospectively reviewed the dual-energy X-ray absorptiometry (DXA) records of older adults at one medical institution in Taipei, Taiwan, from January 1 2019, to December 31, 2020. The inclusion criteria for the eligible population were: 1) aged 60 and older, 2) underwent a full-body DXA scan, and 3) performed hand grip measurements. Patients with duplicate results (*N* = 187), incomplete records (*N* = 205), a history of stroke (*N* = 94), and other neurological diseases (*N* = 11), as well as participants less than 60 years of age (*N* = 192) were excluded. This led to a sample population of 907 participants (Fig. 1).

### Anthropometric measurements

Body weight was determined using an electronic scale, and body height was measured using a fixed stadiometer. BMI was calculated as weight in kilograms divided by height in meters squared (kg/m2).

### Muscle mass measurements

Whole-body lean mass was measured using DXA (Lunar Prodigy, version 9.1; GE Healthcare, Madison, WI). All scans were performed by experienced technicians certified by the International Society for Clinical Densitometry, and all protocols and procedures were strictly followed. Total and regional (trunk, upper extremities, and lower extremities) lean muscle mass were determined using the DXA software. Appendicular skeletal muscle mass (ASM; kg) was calculated by summing the lean masses of the four extremities. The appendicular skeletal muscle index (ASMI) was calculated as ASM in kilograms divided by height in meters squared (kg/m2).

### Handgrip strength measurement

Handgrip strength (HGS, kg) was measured using a dynamometer (Exacta™; North Coast Medical, United States). Three measurements from each hand were obtained, and the maximum value of the dominant hand was recorded for analysis.

### Statistical analysis

Excel and SPSS software were used for data entry. Results are presented as the mean ± the standard deviation (SD). Participants were divided into one of three groups according to age by decade. These groups included those aged 60–69 years, 70–79 years, and 80 years and above. One -way ANOVA was performed to identify significant differences in lean muscle mass and HGS across age groups and sexes. Two-way ANOVA was performed to identify significant differences in the relative declines of regional muscle mass across age and sex. The Bonferroni adjustment was used for post hoc analysis. The two-tailed Pearson test was performed to demonstrate the correlation between HGS decline and regional muscle mass declines. Statistical analyses were performed using PASW Statistics version 18.0 (SPSS Inc., Chicago, IL), and *p* < 0.05 was considered statistically significant.

## Results

Of the 907 participants (Table 1), there were 239 men (mean age, 74.80 ± 9.43) and 668 women (mean age, 72.93 ± 9.09). There were no obese participants, and 95% were right-hand dominant. HGS and regional muscle mass analysis showed a consistent decline after 60 years of age in both sexes (Table 2). Men had greater HGS and lean muscle mass compared to women in all age groups. In the 60–69 age group, men had a mean HGS of 33.78 ± 8.89 kg, while women had a mean HGS of 22.27 ± 4.91 kg.

**Table 1:** Characteristics of the study participants

	Men	Women
Number	239	668
Age	74.80 ± 9.43	72.93 ± 9.09
Height (cm)	165.34 ± 6.49	153.01 ± 6.00
Weight (kg)	65.24 ± 10.73	55.51 ± 9.36
BMI (kg/m^2^)	23.83 ± 3.48	23.72 ± 3.90
**Lean mass**		
Upper extremities (kg)	4.74 ± 0.94	3.27 ± 0.62
Lower extremities (kg)	13.67 ± 2.44	10.41 ± 1.74
Trunk (kg)	21.76 ± 2.88	16.61 ± 2.32
Total lean mass (kg)	43.63 ± 5.81	33.16 ± 4.32
ASM (kg)	18.41 ± 3.18	13.68 ± 2.22
**Handgrip**		
Dominant hand (Right,%)	229 (95.0%)	648 (95.7%)
Grip strength (kg)	28.81 ± 9.87	19.19 ± 6.17

*ASM* Appendicular skeletal muscle

**Table 2:** Age-related changes in lean muscle mass and hand grip strength

	Grip strength	Upper extremities	Lower extremities	Trunk lean mass	Total lean mass	ASM	ASMI
**Men**							
60–69 yrs	33.78 ± 8.89	5.31 ± 0.88	14.77 ± 2.30	23.05 ± 2.63	46.68 ± 5.20	20.09 ± 2.90	7.15 ± 0.93
70–79 yrs	30.58 ± 8.52	4.67 ± 0.80	13.56 ± 2.39	21.52 ± 2.92	43.14 ± 5.67	18.23 ± 3.04	6.68 ± 0.91
80+ yrs	21.32 ± 7.76	4.19 ± 0.77	12.58 ± 2.11	20.60 ± 2.56	40.80 ± 5.02	16.77 ± 2.71	6.28 ± 0.91
*p*-value^a^	< 0.001	< 0.001	< 0.001	< 0.001	< 0.001	< 0.001	< 0.001
**Women**							
60–69 yrs	22.27 ± 4.91	3.40 ± 0.63	10.99 ± 1.73	17.01 ± 2.39	34.32 ± 4.49	14.39 ± 2.24	5.99 ± 0.92
70–79 yrs	19.19 ± 5.26	3.28 ± 0.58	10.31 ± 1.55	16.46 ± 2.17	32.89 ± 3.95	13.59 ± 1.97	5.85 ± 0.83
80+ yrs	13.93 ± 5.44	3.05 ± 0.58	9.53 ± 1.57	16.08 ± 2.22	31.45 ± 3.77	12.58 ± 1.98	5.59 ± 0.88
*p*-value^a^	< 0.001	< 0.001	< 0.001	< 0.001	< 0.001	< 0.001	< 0.001

*ASM* Appendicular skeletal muscle, *ASMI* Appendicular skeletal muscle index

^a^Differences across age-groups were determined by one-way ANOVA

**Fig. 2:**
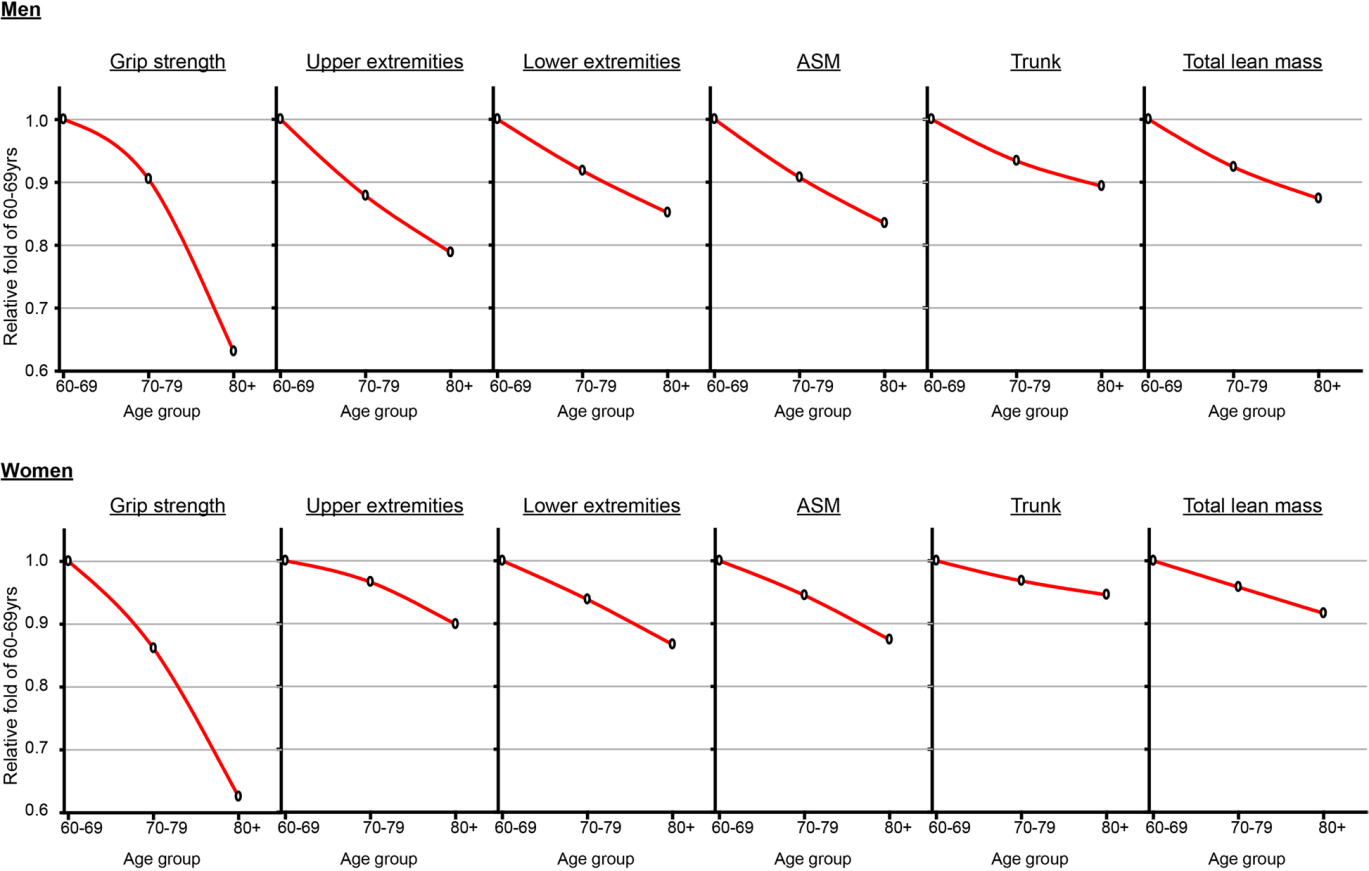
Decline in lean mass and grip strength

The rates of decline per decade in HGS were similar in men (12.30%) and in women (12.48%) (*p* = 0.361, Table 3 and Table 4). The relative decline in total lean muscle mass from the baseline at age 60–69 years old was greater in men (4.20%) than in women (2.79%) (*p* = 0.005, Table 3, Table 4, and Fig. 2). The rates of decline per decade in upper extremity muscle mass, lower extremity muscle mass, and trunk muscle mass were 7.06, 4.95, 3.54% respectively, in men, and 3.36, 4.44, 1.81%, respectively, in women. The relative declines, with the exception of the lower extremities, were significantly different between men and women. However, only decline in the upper extremities correlated significantly with the sex*age interaction variable (Table 4). The relative declines in muscle mass differed significantly by regional body part (upper extremity, lower extremity, and trunk) in both males and females during the aging process (both *p* < 0.001, Table 5). The rate of decline in upper extremity muscle mass was greater than those of other musculature in men (*p* < 0.05 compared to lower extremity mass, and *p* < 0.001 compared to trunk mass), while the rate of decline in lower extremity muscle mass was greater than those of other musculature in women (*p* = 0.233 compared to upper extremity mass, and *p* < 0.01 compared to trunk mass) (see Additional file 1).

**Table 3:** Decline in lean mass and hand grip strength by age

	Grip strength	Upper extremities	Lower extremities	Trunk lean mass	Total lean mass	ASM	ASMI
**Men**							
60–69 yrs	1.00	1.00	1.00	1.00	1.00	1.00	1.00
70–79 yrs	0.91	0.88	0.92	0.93	0.92	0.91	0.93
80+ yrs	0.63	0.79	0.85	0.89	0.87	0.83	0.88
**Women**							
60–69 yrs	1.00	1.00	1.00	1.00	1.00	1.00	1.00
70–79 yrs	0.86	0.97	0.94	0.97	0.96	0.94	0.98
80+ yrs	0.63	0.90	0.87	0.95	0.92	0.87	0.93

*ASM* Appendicular skeletal muscle, *ASMI* Appendicular skeletal muscle index

**Table 4:** The effects of sex and age on the relative declines of the measured parameters

	Sex		Age		Sex * Age	
	F	***P***-value	F	***P***-value	F	***P***-value
**Grip strength**	0.836	0.361	145.840	< 0.001	0.597	0.551
**Upper extremities**	24.296	< 0.001	47.801	< 0.001	6.986	0.001
**Lower extremities**	1.059	0.304	50.745	< 0.001	0.304	0.738
**Trunk lean mass**	8.365	0.004	22.657	< 0.001	2.445	0.087
**Total lean mass**	7.919	0.005	45.247	< 0.001	2.126	0.120
**ASM**	5.381	0.021	57.940	< 0.001	1.428	0.240

**Table 5:** Relative decline of muscle mass in different regional body parts during aging

	Regional body part		Age		Regional body part * Age	
	F	***P***-value	F	***P***-value	F	***P***-value
**Men**	8.389	< 0.001	69.984	< 0.001	2.711	0.029
**Women**	8.447	< 0.001	64.310	< 0.001	3.880	.004

In males overall, HGS significantly correlated with upper (*r* = 0.576, *p < 0.001*) and lower (*r* = 0.523, *p < 0.001*) extremity muscle mass. In females overall, the correlation between HGS and upper extremity muscle mass (*r* = 0.262, *p < 0.001*) and HGS and lower extremity muscle mass (*r* = 0.364, *p < 0.001*) were less strong, though also statistically significant (Table 6). When examining these relationships across each of the age subgroups, the correlations between HGS and upper and lower extremity muscle mass remained statistically significant in males in all three subgroups. In females however, the relationships were less strong and became weaker with increasing age, though for the most part remained statistically significant, with the exception of the relationship between HGS and upper extremity mass in 70–79 yrs., which did not reach statistical significance (Table 6).

**Table 6:** Age-dependent correlations between regional lean muscle decline and hand grip strength decline

Grip strength	Upper extremities	Lower extremities	Trunk lean mass	Total lean mass
**> = 60 yrs**				
Men	0.576***	0.523***	0.313***	0.473***
Women	0.262***	0.364***	0.110**	0.258***
**60–69 yrs**				
Men	0.436***	0.332**	0.098	0.294**
Women	0.189**	0.249***	0.053	0.164**
**70–79 yrs**				
Men	0.452***	0.537***	0.097	0.348**
Women	0.139	0.232**	−0.010	0.118
**80+ yrs**				
Men	0.481***	0.425***	0.415***	0.435***
Women	0.173*	0.178*	0.014	0.108

^*^p < 0.05

^**^p < 0.01

^***^p < 0.001

## Discussion

Although previous research has demonstrated a correlation between muscle strength and regional muscle mass, few studies have investigated the relationship between HGS and muscle mass in older adults. This study compares the rates of decline in HGS and muscle mass in men and women and determines the relationship between HGS and upper and lower extremity muscle mass in older Asian adults.

We found that men had greater HGS and lean mass compared to women in all age groups. Both muscle strength and mass declined at a faster rate in men than in women. A Thai study that included 491 older adult participants with a mean age of 69.15 ± 6.86 years found that the mean HGS in males (24.93 ± 7.45) was significantly higher than in females (16.17 ± 4.82) [19]. Both sexes also experienced a significant decline in HGS with age. However, the mean HGS for both men and women in the Thai study were lower than those found in our study for both sexes in the 60–69 and 70–79 year age groups, respectively. The Thai study also recorded certain preexisting medical conditions that may potentially have contributed to the lower HGS scores in their participants, including 26.1% with osteoporosis and 20.4% with osteoarthritis, among others. In addition, other lifestyle factors were also assessed, such as active smoking habits and regular medication intake. In a recent Singaporean study, the mean HGS in 2043 adults aged 60 years and above also declined with increasing age [14]. HGS in these Singaporeans was low compared to those in Western and some other Asian countries, though was comparable to the participants in our study [14, 15, 20].

Our study revealed that musculature in the upper extremities declines faster than elsewhere in men, whereas in women, it is lower extremity musculature that declines most rapidly. Janssen et al. found that men had significantly more skeletal muscle than women (*p* < 0.001), and the difference in skeletal muscle between sexes was greatest in the upper body. In addition, skeletal muscle mass decreased with increasing age, especially after 50 years of age [21]. Recently, Kolodziej et al. found that the annual declines in muscle strength and function were greater than the decline in appendicular skeletal muscle mass in 313 older adults aged 50–83 [12].

In our study, HGS significantly correlated with lean muscle mass in the upper and lower extremities in men, and also for the most part, though more weakly, in women. A 2018 U.S. study that included 2994 men aged 65 and above found that appendicular lean muscle mass was a better indicator of overall health compared to grip strength and muscle power [22]. An earlier study based in Canada confirmed an association between muscle strength and lean body mass and, in men, demonstrated a strong association between handgrip strength and the appendicular lean body mass index [23]. Data from our study revealed similar findings in an older adult Asian population. In addition, because HGS strongly correlated with upper and lower extremity muscle mass in men, HGS can potentially serve as a proxy for muscle mass in clinical settings, at least in men.

Our results suggest that in contrast to trunk muscle mass, extremity muscle mass declines more rapidly with age. Wilkinson et al proposed that the muscle loss that occurs with age may be caused by anabolic resistance due to decreased physical activity and food intake, factors that help regulate the ongoing muscle protein synthesis and breakdown needed to maintain muscle mass [24]. While food intake should have a similar effect on both the muscles of the trunk and the extremities, physical activity with respect to certain regional muscles can vary significantly, especially with age. Therefore, this difference between rates of decline may be at least partially explained by muscle use. Older adults tend to develop arthritic changes in their extremities and consequently use their extremity muscles less often. Other conditions that occur with age such as stroke and neurodegenerative disorders likely also more greatly affect the usage of extremity musculature than central musculature as well. Conversely, the trunk musculature is not nearly as severely affected by degenerative conditions and may be used in a normal or near normal fashion as a person ages. In addition, Wilkinson et al observed that with age, there is a greater tendency for muscles to resist anabolic stimuli, such as mechanical sensing and feeding, though reasons for this have not been thoroughly explored [24]. This resistance to anabolic stimuli likely compounds the loss in extremity musculature that occurs with disuse. When the muscle attempts to rebuild itself following a period of disuse, it encounters significant resistance. This results in an even weaker muscle, which is even less likely to be used and ultimately a vicious cycle of continued muscle loss.

While extremity muscle mass declines faster than trunk mass in older adults, the type of extremity mass that declines fastest differs by gender. Our finding that upper extremity muscle mass declines faster in men, while lower extremity muscle mass declines faster in women may be secondary to a number of phenomena. Janssen et al found that weight and height accounted for 50% of the variance in muscle mass in men and women, noting that muscle mass decreased with increasing body weight, therefore suggesting increased levels of fat [21]. The study also found that men have more absolute muscle mass than women in the upper body [21]. Therefore, men may simply have relatively greater amounts of upper extremity muscle mass to begin with and therefore more to lose, resulting in a faster rate of decline. Diminished muscle mass and strength may also be the result of the sharp hormone changes that occur with age. Cauley et al found that in addition to age, loss of grip strength in postmenopausal women may be due to depleted levels of ovarian estrogen, among other factors [25]. A 2020 study found that grip strength and testosterone levels were inversely correlated in men, noting that the correlation was lower among obese individuals compared to non-obese individuals [26]. While hormonal changes in older adults undoubtedly play a role in the rates of muscle decline, they would be expected to have a similar effect on the musculature throughout the entire body and not favor a particular area. However, hormones can certainly have more targeted effects, and the density of receptors in a certain region will define their impact. Perhaps the estrogen and testosterone receptor densities in the lower and upper extremity musculature of women and men respectively define their rates of muscle loss. Hormones may also compound the muscle loss that occurs secondary to other reasons. For example, if muscle loss begins to occur in the upper extremities of a male for an unknown reason, changes in testosterone levels may accelerate this loss, contrasting it more starkly with the muscle loss that occurs in other regions. Much remains unknown about the role of hormones in muscle loss in older adults. More research is necessary to elucidate the precise effects of hormones on muscle strength and mass.

It is not surprising that total muscle mass decline correlates with HGS, though our finding that regional muscle mass decline also correlates with HGS is interesting. Of further interest is the fact that this correlation is stronger in men, particularly with age. The reason for such a difference across genders is unclear, but we can look to the same factors that may help explain the differences across gender in rates of upper extremity and lower extremity muscle loss, namely baseline upper extremity muscle bulk and hormones. Again, men at baseline tend to have more upper body muscle bulk [22]. Because upper extremity musculature is used to perform HGS measurements and men have greater muscle mass in this area, they would be expected to decline together more closely. In addition, our finding that men experience declines in upper extremity musculature most rapidly suggests that a corresponding drop in HGS scores occurs with the muscle loss. Again, hormones likely also play a role, though that role is not precisely clear [26]. Similar to their potential role in rates of extremity muscle loss, hormones may exacerbate the losses in HGS that occur more severely in males or perhaps hormones may specifically target the upper extremity musculature of males. Again, more research is needed to identify the role of hormones in muscle loss.

### Limitations of the study

This retrospective cross-sectional study offers strong evidence of the relationship between HGS and muscle mass in older Asian adults, however a prospective longitudinal study would offer more rigorous evidence in support of this relationship. Future research examining this relationship prospectively would be useful, though the difficulties with regards to recruiting participants for such a study may make this challenging. Further, the external validity of our results is limited by the fact that all study participants were recruited from a single institution. Future research that includes participants from multiple institutions in multiple areas (e.g., urban and rural) would increase the generalizability of the results.

## Conclusion

In conclusion, HGS and all categories of lean muscle mass were greater in men than in women across all age groups. Muscle mass and HGS decline with advancing age in both older men and women though the correlation is stronger in men than in women, especially in the upper extremities. HGS measurements are an accurate proxy for muscle mass in older Asian adults, particularly in males.

## Supplementary Information

**Additional file 1.** Results of post hoc Bonferroni test for relative declines in different regional body parts.

## Data Availability

The datasets used and/or analyzed during the current study are available from the corresponding author on reasonable request.

## Abbreviations

HGS: Hand grip strength
DXA: Dual-energy X-ray absorptiometry
ASM: Appendicular skeletal muscle mass
ASMI: Appendicular skeletal muscle index
AWGS: The Asian Working Group for Sarcopenia
SD: Standard deviation

## References

[1] Gale CR, Martyn CN, Cooper C, Sayer AA (2007). Grip strength, body composition, and mortality. Int J Epidemiol.

[2] Sasaki H, Kasagi F, Yamada M, Fujita S (2007). Grip strength predicts cause-specific mortality in middle-aged and elderly persons. Am J Med.

[3] Ruiz JR, Sui X, Lobelo F, Morrow JR, Jackson AW, Sjostrom M, Blair SN (2008). Association between muscular strength and mortality in men: prospective cohort study. BMJ.

[4] Rantanen T, Harris T, Leveille SG, Visser M, Foley D, Masaki K, Guralnik JM (2000). Muscle strength and body mass index as long-term predictors of mortality in initially healthy men. J Gerontol A Biol Sci Med Sci.

[5] Al Snih S, Markides KS, Ray L, Ostir GV, Goodwin JS (2002). Handgrip strength and mortality in older Mexican Americans. J Am Geriatr Soc.

[6] Newman AB, Kupelian V, Visser M, Simonsick EM, Goodpaster BH, Kritchevsky SB, Tylavsky FA, Rubin SM, Harris TB (2006). Strength, but not muscle mass, is associated with mortality in the health, aging and body composition study cohort. J Gerontol A Biol Sci Med Sci.

[7] Rantanen T, Masaki K, He Q, Ross GW, Willcox BJ, White L (2012). Midlife muscle strength and human longevity up to age 100 years: a 44-year prospective study among a decedent cohort. Age (Dordr).

[8] Ling CH, Taekema D, de Craen AJ, Gussekloo J, Westendorp RG, Maier AB (2010). Handgrip strength and mortality in the oldest old population: the Leiden 85-plus study. CMAJ.

[9] Volaklis KA, Halle M, Meisinger C (2015). Muscular strength as a strong predictor of mortality: a narrative review. Eur J Intern Med.

[10] FitzGerald SJ, Barlow CE, Kampert JB, Morrow JR, Jackson AW, Blair SN (2004). Muscular fitness and all-cause mortality: prospective observations. J Phys Act Health.

[11] Goodpaster BH, Park SW, Harris TB, Kritchevsky SB, Nevitt M, Schwartz AV, Simonsick EM, Tylavsky FA, Visser M, Newman AB (2006). The loss of skeletal muscle strength, mass, and quality in older adults: the health, aging and body composition study. J Gerontol A Biol Sci Med Sci.

[12] Kolodziej M, Ignasiak Z, Ignasiak T (2021). Annual changes in appendicular skeletal muscle mass and quality in adults over 50 y of age, assessed using bioelectrical impedance analysis. Nutrition.

[13] Sui SX, Holloway-Kew KL, Hyde NK, Williams LJ, Tembo MC, Mohebbi M, Gojanovic M, Leach S, Pasco JA (2020). Handgrip strength and muscle quality in Australian women: cross-sectional data from the Geelong osteoporosis study. J Cachexia Sarcopenia Muscle.

[14] Ong HL, Abdin E, Chua BY, Zhang Y, Seow E, Vaingankar JA, Chong SA, Subramaniam M (2017). Hand-grip strength among older adults in Singapore: a comparison with international norms and associative factors. BMC Geriatr.

[15] Bohannon RW (2019). Grip strength: an indispensable biomarker for older adults. Clin Interv Aging.

[16] Wind AE, Takken T, Helders PJ, Engelbert RH (2010). Is grip strength a predictor for total muscle strength in healthy children, adolescents, and young adults?. Eur J Pediatr.

[17] Choe YR, Jeong JR, Kim YP (2020). Grip strength mediates the relationship between muscle mass and frailty. J Cachexia Sarcopenia Muscle.

[18] Shimokata H, Ando F, Yuki A, Otsuka R (2014). Age-related changes in skeletal muscle mass among community-dwelling Japanese: a 12-year longitudinal study. Geriatr Gerontol Int.

[19] Horpibulsuk J, Nutkhum W, Jongjol P (2019). Handgrip strength of community-dwelling elderly in Nakhon Ratchasima Province, Thailand. Chiang Mai Med J.

[20] Wu SW, Wu SF, Liang HW, Wu ZT, Huang S (2009). Measuring factors affecting grip strength in a Taiwan Chinese population and a comparison with consolidated norms. Appl Ergon.

[21] Janssen I, Heymsfield SB, Wang ZM, Ross R (2000). Skeletal muscle mass and distribution in 468 men and women aged 18-88 yr. J Appl Physiol (1985).

[22] Chalhoub D, Boudreau R, Greenspan S, Newman AB, Zmuda J, Frank-Wilson AW, Nagaraj N, Hoffman AR, Lane NE, Stefanick ML (2018). Associations between lean mass, muscle strength and power, and skeletal size, density and strength in older men. J Bone Miner Res.

[23] Barbat-Artigas S, Plouffe S, Pion CH, Aubertin-Leheudre M (2013). Toward a sex-specific relationship between muscle strength and appendicular lean body mass index?. J Cachexia Sarcopenia Muscle.

[24] Wilkinson DJ, Piasecki M, Atherton PJ (2018). The age-related loss of skeletal muscle mass and function: measurement and physiology of muscle fibre atrophy and muscle fibre loss in humans. Ageing Res Rev.

[25] Cauley JA, Petrini AM, LaPorte RE, Sandler RB, Bayles CM, Robertson RJ, Slemenda CW (1987). The decline of grip strength in the menopause: relationship to physical activity, estrogen use and anthropometric factors. J Chronic Dis.

[26] Chiu HT, Shih MT, Chen WL (2020). Examining the association between grip strength and testosterone. Aging Male.

