# Supplementary material for: Correlation between hand grip strength and regional muscle mass in older Asian adults: an observational study
Source: BMC Geriatr. 2022 Mar 15;22:206. doi: 10.1186/s12877-022-02898-8 (PMC8922763; doi:10.1186/s12877-022-02898-8)
Supplement: Supplementary file 1 — Additional file 1. Results of post hoc Bonferroni test for relative declines in different regional body parts. [file 12877_2022_2898_MOESM1_ESM.docx]

**Additional File 1. Results of post hoc Bonferroni test for relative declines in different regional body parts**

|  | **Comparison** | |  |  |  | **95% confidence interval** | |
| --- | --- | --- | --- | --- | --- | --- | --- |
| **Sex** | **Regional body part** | **Regional body part** | **Mean difference** | **Standard error** | **p-value** | **lower** | **upper** |
| Men | Upper extremity | Lower extremity | -0.033 | 0.013 | 0.035 | -0.065 | -0.002 |
|  |  | Trunk | -0.051 | 0.013 | 0.000 | -0.083 | -0.020 |
|  | Lower extremity | Upper extremity | 0.033 | 0.013 | 0.035 | 0.002 | 0.065 |
|  |  | Trunk | -0.018 | 0.013 | 0.501 | -0.050 | 0.013 |
|  | Trunk | Upper extremity | 0.051 | 0.013 | 0.000 | 0.020 | 0.083 |
|  |  | Lower extremity | 0.018 | 0.013 | 0.501 | -0.013 | 0.050 |
| Women | Upper extremity | Lower extremity | 0.015 | 0.008 | 0.233 | -0.005 | 0.035 |
|  |  | Trunk | -0.014 | 0.008 | 0.306 | -0.034 | 0.006 |
|  | Lower extremity | Upper extremity | -0.015 | 0.008 | 0.233 | -0.035 | 0.005 |
|  |  | Trunk | -0.029 | 0.008 | 0.002 | -0.049 | -0.009 |
|  | Trunk | Upper extremity | 0.014 | 0.008 | 0.306 | -0.006 | 0.034 |
|  |  | Lower extremity | 0.029 | 0.008 | 0.002 | 0.009 | 0.049 |
